# Supplementary material for: Approaches to quantify the contribution of multiple anemia risk factors in children and women from cross-sectional national surveys
Source: PLOS Glob Public Health. 2022 Oct 13;2(10):e0001071. doi: 10.1371/journal.pgph.0001071 (PMC10022287; doi:10.1371/journal.pgph.0001071)
Supplement: S1 Table — (DOCX) [file pgph.0001071.s001.docx]

**S1 Table. Estimated attributable fractions of anemia associated with various risk factors in preschool children**

|  | Country | Uni. OR | Adj. OR | Uni. PR | Adj. PR | Adj. PR (Zhang-Yu) | Adj. PR (Kleinman-Norton) | Average AF | Adj. Average AF |
| --- | --- | --- | --- | --- | --- | --- | --- | --- | --- |
| Inflammation | Côte d'Ivoire | 49.3% | 42.9% | 18.2% | 15.3% | 16.1% | 14.4% | 17.4% | 14.9% |
|  | Cameroon | 57.7% | 43.0% | 30.2% | 19.8% | 23.6% | 16.7% | 18.7% | 16.5% |
|  | Liberia | 31.7% | 23.7% | 13.9% | 10.2% | 10.7% | 9.6% | 9.2% | 9.4% |
|  | Malawi | 36.3% | 11.9% | 26.6% | 7.4% | 9.1% | 6.7% | 7.3% | 6.2% |
|  | Azerbaijan | 21.3% | 28.3% | 15.5% | 19.0% | 20.1% | 18.3% | 15.4% | 16.1% |
|  | Cambodia | 9.2% | 4.3% | 4.4% | 2.0% | 2.1% | 1.8% | 3.0% | 3.2% |
|  | Laos | 5.6% | -0.8% | 3.3% | -0.6% | -0.5% | -0.4% | 3.1% | -0.4% |
|  | Afghanistan | 13.7% | 13.6% | 7.4% | 7.2% | 7.4% | 6.7% | 7.1% | 7.2% |
|  | Bangladesh | 21.6% | 28.8% | 13.4% | 16.9% | 17.2% | 14.8% | 15.0% | 14.8% |
|  | Nepal | 18.4% | 18.7% | 14.3% | 12.2% | 14.4% | 12.3% | 14.2% | 12.0% |
| Iron | Côte d'Ivoire | 15.0% | 8.2% | 4.1% | 2.3% | 2.4% | 2.1% | 4.4% | 2.3% |
| Deficiency | Cameroon | 29.2% | 47.3% | 12.8% | 17.5% | 18.5% | 16.3% | 21.2% | 18.8% |
|  | Liberia | 15.7% | 26.3% | 6.4% | 10.2% | 10.2% | 10.5% | 12.1% | 11.4% |
|  | Malawi | 19.6% | 21.6% | 12.1% | 12.7% | 13.1% | 10.6% | 14.6% | 10.5% |
|  | Azerbaijan | 29.7% | 30.3% | 20.0% | 19.0% | 20.4% | 19.2% | 22.5% | 20.7% |
|  | Cambodia | 27.6% | -- | 3.6% | -- | -- | -- | 4.2% | 3.3% |
|  | Laos | 34.0% | 20.8% | 16.9% | 9.5% | 11.5% | 10.4% | 17.6% | 11.0% |
|  | Afghanistan | 16.8% | 14.1% | 9.0% | 7.1% | 7.7% | 6.9% | 7.2% | 6.4% |
|  | Bangladesh | 31.5% | 33.4% | 14.3% | 14.3% | 14.9% | 14.0% | 20.5% | 19.1% |
|  | Nepal | 51.7% | 45.6% | 39.1% | 32.0% | 35.1% | 32.4% | 36.1% | 32.7% |
| Vitamin A | Côte d'Ivoire | 0.2% | -0.1% | 0.0% | 0.0% | 0.0% | 0.0% | -0.1% | 0.0% |
| Deficiency | Cameroon | 9.7% | 10.8% | 3.4% | 1.7% | 3.7% | 2.8% | 2.5% | 3.1% |
|  | Liberia | 2.8% | 2.1% | 0.9% | 0.6% | 0.7% | 0.7% | 0.6% | 0.7% |
|  | Malawi | 6.5% | 6.7% | 3.8% | 3.5% | 3.9% | 3.1% | 2.6% | 2.5% |
|  | Azerbaijan | 5.8% | 2.0% | 3.8% | 1.1% | 1.4% | 1.2% | 1.4% | 0.8% |
|  | Cambodia | 1.2% | 2.2% | 0.5% | 0.7% | 0.9% | 0.8% | -0.1% | 0.3% |
|  | Afghanistan | 24.4% | 19.3% | 14.2% | 10.9% | 11.5% | 10.5% | 11.4% | 11.4% |
|  | Bangladesh | 4.0% | 1.9% | 2.2% | 1.0% | 1.1% | 0.9% | 2.1% | 2.2% |
|  | Nepal | 1.1% | -0.5% | 0.9% | -0.4% | -0.4% | -0.3% | -0.4% | -0.3% |
| B12 | Cameroon | 16.9% | 15.2% | 6.1% | 3.9% | 5.7% | 4.2% | 4.9% | 4.2% |
| Deficiency | Malawi | 8.6% | 6.0% | 4.0% | 2.0% | 3.2% | 2.4% | 2.5% | 2.1% |
|  | Cambodia | 0.8% | -- | 0.3% | -- | -- | -- | 0.3% | -0.1% |
|  | Nepal | 1.1% | -- | 0.8% | -- | -- | -- | -- | -- |
| Folate | Cameroon | 14.0% | 8.8% | 4.2% | 2.3% | 3.1% | 2.3% | 2.4% | 2.6% |
| Deficiency | Malawi | 0.4% | 0.2% | 0.2% | 0.1% | 0.1% | 0.1% | 0.2% | 0.2% |
|  | Cambodia | 5.6% | 10.7% | 2.1% | 3.4% | 3.2% | 2.9% | 3.0% | 3.5% |
|  | Nepal | -0.4% | -- | -0.4% | -- | -- | -- | -0.4% | -0.2% |
| Malaria | Côte d'Ivoire | 14.8% | 7.4% | 3.7% | 1.8% | 2.1% | 1.8% | 2.4% | 2.0% |
|  | Cameroon | 57.0% | 58.1% | 20.4% | 16.5% | 20.7% | 17.5% | 18.5% | 18.1% |
|  | Liberia | 34.1% | 39.8% | 11.2% | 12.8% | 12.5% | 12.3% | 10.4% | 11.2% |
|  | Malawi | 48.1% | 61.3% | 29.7% | 35.2% | 35.7% | 33.3% | 29.1% | 30.7% |
| Blood | Malawi | 9.0% | 20.0% | 6.3% | 11.4% | 13.5% | 11.0% | 9.1% | 8.8% |
| Disorders | Cambodia | 50.9% | 46.6% | 30.9% | 25.4% | 28.4% | 24.5% | 30.8% | 24.7% |
|  | Nepal | 5.8% | 4.7% | 4.2% | 2.8% | 3.5% | 2.9% | 2.2% | 2.3% |

OR=odds ratio; PR=prevalence ratio. Average AF considers all exposure variables. Adjusted average AF considers all exposure variables and adjusts for age, sex, and SES.
