# Supplementary material for: Approaches to quantify the contribution of multiple anemia risk factors in children and women from cross-sectional national surveys
Source: PLOS Glob Public Health. 2022 Oct 13;2(10):e0001071. doi: 10.1371/journal.pgph.0001071 (PMC10022287; doi:10.1371/journal.pgph.0001071)
Supplement: S2 Table — (DOCX) [file pgph.0001071.s002.docx]

**S2 Table. Estimated attributable fractions of anemia associated with various risk factors in women of reproductive age**

|  | Country | Uni. OR | Adj. OR | Uni. PR | Adj. PR | Adj. PR (Zhang-Yu) | Adj. PR (Kleinman-Norton) | Average AF | Adj. Average AF |
| --- | --- | --- | --- | --- | --- | --- | --- | --- | --- |
| Inflammation | Côte d'Ivoire | 7.3% | 4.8% | 3.6% | 2.5% | 2.4% | 2.3% | 2.5% | 2.2% |
|  | Cameroon | 14.7% | 22.2% | 8.2% | 10.1% | 11.4% | 9.3% | 9.7% | 9.8% |
|  | Liberia | 16.2% | 16.4% | 9.2% | 8.6% | 9.4% | 8.5% | 8.3% | 8.4% |
|  | Malawi | 6.8% | 3.7% | 4.9% | 2.0% | 2.8% | 2.3% | 2.6% | 2.5% |
|  | Azerbaijan | 3.5% | 7.5% | 2.1% | 4.0% | 4.4% | 3.5% | 2.1% | 3.1% |
|  | Cambodia | -8.1% | -7.1% | -4.6% | -3.9% | -4.0% | -3.9% | -4.1% | -4.3% |
|  | Laos | -0.6% | 0.1% | -0.3% | 0.0% | 0.0% | 0.0% | 0.0% | 0.0% |
|  | Afghanistan | 11.1% | 12.0% | 6.0% | 6.2% | 6.4% | 5.7% | 5.4% | 5.6% |
|  | Bangladesh | 8.6% | 8.6% | 5.8% | 4.6% | 5.8% | 5.3% | 5.4% | 5.3% |
|  | Nepal | 4.8% | 5.0% | 2.8% | 2.7% | 3.0% | 2.6% | 2.6% | 2.7% |
|  | Pakistan | 0.1% | -0.2% | 0.0% | -0.2% | -0.1% | -0.1% | -0.2% | -0.1% |
| Iron | Côte d'Ivoire | 26.4% | 26.9% | 10.9% | 11.0% | 11.1% | 10.9% | 10.4% | 10.8% |
| Deficiency | Cameroon | 61.6% | 66.9% | 27.1% | 29.6% | 28.4% | 28.0% | 29.8% | 29.7% |
|  | Liberia | 37.2% | 37.4% | 22.2% | 21.5% | 22.3% | 21.5% | 22.4% | 22.0% |
|  | Malawi | 42.2% | 47.2% | 25.5% | 27.3% | 27.6% | 26.4% | 27.9% | 28.1% |
|  | Azerbaijan | 71.0% | 70.0% | 46.8% | 45.3% | 46.2% | 44.3% | 45.1% | 45.1% |
|  | Cambodia | 15.6% | -- | 3.0% | -- | -- | -- | 2.5% | 2.5% |
|  | Laos | 41.8% | 41.9% | 20.5% | 20.4% | 20.6% | 20.4% | 19.7% | 19.7% |
|  | Afghanistan | 31.5% | 30.6% | 17.2% | 16.0% | 16.8% | 15.2% | 16.2% | 15.3% |
|  | Bangladesh | 24.1% | 26.6% | 12.0% | 11.8% | 12.7% | 12.4% | 13.6% | 14.3% |
|  | Nepal | 44.5% | 39.7% | 24.2% | 21.0% | 22.2% | 21.4% | 25.3% | 22.7% |
|  | Pakistan | 36.4% | 35.9% | 24.8% | 23.1% | 24.5% | 23.5% | 21.4% | 21.9% |
| Vitamin A | Côte d'Ivoire | 6.2% | 3.4% | 2.8% | 1.3% | 1.7% | 1.6% | 1.7% | 1.4% |
| Insufficiency | Cameroon | 6.4% | -2.6% | 3.9% | -1.6% | -1.8% | -1.3% | -1.3% | -1.3% |
|  | Liberia | 18.8% | 15.6% | 10.8% | 8.3% | 9.3% | 8.4% | 8.0% | 8.0% |
|  | Malawi | 17.8% | 16.5% | 12.6% | 10.6% | 11.7% | 9.7% | 8.3% | 8.3% |
|  | Azerbaijan | 23.9% | 15.1% | 7.1% | 3.3% | 5.6% | 4.5% | 5.2% | 4.9% |
|  | Cambodia | 8.2% | 7.6% | 3.4% | 3.2% | 3.3% | 3.3% | 2.2% | 2.2% |
|  | Laos | 18.6% | 16.7% | 9.6% | 8.1% | 8.7% | 8.2% | 8.9% | 8.2% |
|  | Afghanistan | 24.2% | 21.4% | 14.3% | 11.8% | 12.8% | 11.4% | 13.6% | 12.2% |
|  | Bangladesh | 11.9% | 6.2% | 8.6% | 3.7% | 4.6% | 4.1% | 2.9% | 3.7% |
|  | Pakistan | 25.4% | 22.6% | 18.1% | 14.6% | 16.4% | 15.1% | 14.6% | 14.5% |
| B12 | Côte d'Ivoire | 7.5% | 2.6% | 3.4% | 1.1% | 1.3% | 1.2% | 1.9% | 1.2% |
| Deficiency | Cameroon | 0.8% | 4.7% | 0.5% | 2.9% | 2.8% | 2.2% | 2.4% | 2.0% |
|  | Malawi | -1.1% | -0.9% | -0.9% | -0.4% | -0.7% | -0.6% | -0.8% | -0.4% |
|  | Azerbaijan | -1.8% | -3.2% | -1.1% | -1.4% | -2.0% | -1.6% | -1.6% | -1.5% |
|  | Cambodia | -0.8% | -- | -0.7% | -- | -- | -- | -0.6% | -0.6% |
|  | Laos | -0.2% | 0.0% | -0.1% | 0.0% | 0.0% | 0.0% | -0.4% | 0.0% |
|  | Bangladesh | -0.2% | -1.1% | -0.1% | -0.5% | -0.8% | -0.8% | -0.5% | -0.7% |
|  | Pakistan | -0.1% | -- | -0.1% | -- | -- | -- | -- | -- |
| Folate | Côte d'Ivoire | -17.1% | -24.5% | -8.1% | -10.8% | -11.7% | -10.8% | -13.9% | -10.8% |
| Deficiency | Cameroon | 13.3% | 7.0% | 7.3% | 1.8% | 4.2% | 3.2% | 3.0% | 3.3% |
|  | Malawi | 2.4% | 1.7% | 1.8% | 1.5% | 1.3% | 1.1% | 1.7% | 1.0% |
|  | Azerbaijan | 1.0% | -- | 0.5% | -- | -- | -- | -0.2% | -0.2% |
|  | Cambodia | 7.2% | 8.1% | 3.6% | 4.0% | 4.0% | 4.0% | 3.5% | 3.7% |
|  | Laos | 6.7% | 6.7% | 3.3% | 3.1% | 3.3% | 3.2% | 3.2% | 3.1% |
|  | Bangladesh | 15.2% | 14.5% | 10.9% | 9.2% | 10.4% | 9.5% | 9.7% | 10.1% |
|  | Pakistan | -1.0% | -- | -0.8% | -- | -- | -- | -1.4% | -1.4% |
| Malaria | Côte d'Ivoire | 5.0% | 4.5% | 1.9% | 1.6% | 1.7% | 1.7% | 1.8% | 1.6% |
|  | Cameroon | 8.9% | 10.2% | 5.0% | 5.1% | 5.6% | 4.4% | 4.7% | 4.4% |
|  | Liberia | 3.8% | 2.5% | 2.4% | 1.5% | 1.6% | 1.5% | 1.3% | 1.2% |
|  | Malawi | 12.7% | 24.4% | 9.1% | 15.8% | 15.8% | 13.3% | 9.8% | 10.6% |

OR=odds ratio; PR=prevalence ratio. Average AF considers all exposure variables. Adjusted average AF considers all exposure variables and adjusts for age, sex, and SES.
